# Dataset of phase-resolved images of internal, corona, and surface partial discharges in electrical generators

**DOI:** 10.1016/j.dib.2023.109992

**Published:** 2023-12-20

**Authors:** Juan David Zorrilla Henao, Alejandro Segura, Alejandro Tenorio, Harold José Diaz, Alejandro Paz

**Affiliations:** a School of Electrical and Electronic Engineering (IEEE), Faculty of Engineering, Universidad del Valle; b Faculty of Engineering, Santiago de Cali University

**Keywords:** Partial discharge, Generator, Internal, Corona, Surface, Data augmentation

## Abstract

This article presents the data collection process for the classification of partial discharges in electrical generators using PNG format images. The data were collected through field measurements on over 40 generators in various locations in Colombia, in addition to utilizing a partial discharge simulator provided by Omicron Energy.

Throughout the collection process, special attention was given to the accuracy and coherence of the images, avoiding deformations and distortions that could impact the nature of partial discharges. Emphasis was placed on achieving high resolution in phase-resolved patterns (PRPD) to effectively correlate them with the adjacent physical phenomenon. The analysis focused on classifying the images according to the type of partial discharge, identifying them as internal, surface, or corona discharges. The obtained pulse patterns are represented in RGB color, which aids in assessing the repeatability of pulses across their distribution.

These data hold potential for the development of pattern classification software for generator monitoring systems. They enable the training and validation of classification algorithms, simplifying the automated detection and analysis of partial discharges in electrical generators. Their applicability extends beyond the electrical industry and can be valuable in other fields requiring complex signal and pattern analysis.

The article highlights the rigorous data collection process and precise analysis conducted to obtain a valuable set of PNG format images for partial discharge classification. These data have significant potential in advancing pattern classification software, driving progress in the monitoring and analysis of electrical generators.

Specifications Table

**Table:** 

Subject	Data Engineering Data Science Artificial Intelligence
Specific subject area	Diagnosis of Insulation in High-Power Rotating Machinery. Pattern Recognition.
Type of data	Image
How the data were acquired	MPD Simulator with Electrodes, Direct Measurement in Generators using MPD 600 Equipment with Coupling Capacitor and Voltage Injection Source CPC100, and TD by OMICRON. Utilization of Image Processing Programming.
Data format	PNG image (570 × 440 pixeles).
Description of data collection	The data were collected through field measurements conducted at hydroelectric power plants and thermal generation facilities spread across the national territory. Additionally, laboratory data were gathered using a simulator and electrodes provided by OMICRON Energy, a specialized manufacturer of insulation measurement equipment for power assets such as transformers and rotating machines.
Data source location	Institution: Universidad del Valle City/Town/Region: Cali, Valle del Cauca Country: Colombia Continent: South America
Data accessibility	Repository Name: Images of Resolved Phase Patterns of Partial Discharges in Electric Generators Data Identification Number: Version 8.0 Direct Data URL: https://data.mendeley.com/datasets/xz4xhrc4yr/8

## 1 Value of the Data

• The dataset of images can be used to classify patterns of partial discharge in electrical generators with voltages greater than 6.6 kV. Early intervention in the machine based on its defect can prevent unexpected shutdowns, ensuring a continuous power supply to the communities served by the generator.
• The dataset of images can serve as training and evaluation data for artificial intelligence systems that require pattern recognition without a predefined form.
• Phase-Resolved Patterns of Defects (PRPD) in power generation units in the country are scarce, making this dataset particularly valuable for research and analysis.
• PRPD data should have been collected in accordance with the parameters and procedures specified in standards such as IEC 60270, IEC 60034, and IEEE 1434, with the approval of an expert, ensuring their reliability and quality.
• Proper filtering of PRPD data has been performed to avoid confusion with images resulting from electromagnetic noise in real-world situations, ensuring that the dataset reflects genuine partial discharge patterns.
• Data augmentation techniques have been scientifically employed to enhance the dataset's richness and diversity, contributing to its usefulness in pattern recognition and machine learning applications.

## 2 Objective

The dataset comprises PNG images generated by the MPD And MI 1.6.7.1 software, representing the phase-resolved pattern of a partial discharge (PRPD). These images have been filtered to remove any irrelevant background information, with no additions to the pattern figure. No noise filtering or other adjustments have been applied to the images. The images do not contain information about the magnitude of partial discharges, as their purpose is to differentiate pattern shapes, not to assess their magnitude or criticality. These aspects depend on the calibration at the time of data capture and the measured object.

A significant portion of the images in the dataset was generated using computational tools based on a series of images directly captured from electrical generators in power generation facilities. These images adhere to parameters set by international standards such as IEC 60034-27 and IEEE 1434, ensuring their compliance with recognized standards.

Table 1 displays the quantity of images categorized based on their download nature and acquisition method. Table 2 provides detailed information about the specimens from which the actual images were captured, and Table 3 illustrates the distribution of images within the dataset, along with their corresponding subfolder breakdown.

**Table 1:** Number of images and their distribution.

Type of DP	Real	Simulated	Data augmentation	Total
Corona	4	5	299	308
Internal	17	5	299	321
Surface	14	2	300	316
Noise	5	0	0	5
Total	40	12	898	**950**

**Table 2:** General information on the source of real images.

Type of DP	Power	Voltage level	Description
Corona	12–44 MVA	13.2–13.8 kV	Units of generation with corona-type discharges in coil heads due to insulation breakdown and heavy contamination in coil heads.
internal	12–220 MVA	13.2–13.8 kV	Generation units, including some thermal units, with mild and moderate delamination defects.
surface	12–44 MVA	13.2–13.8 kV	Generation units, including some thermal ones, experiencing partial discharges due to high contamination, insulation breakdowns, and provisional insulation repairs in coil heads and in the area between clamping rings and the core.
Noise	All	13.2–13.8 kV	In all measurements, noise is generally measured to determine the initial baseline magnitude for PD (Partial Discharge) measurements.

**Table 3:** Dataset file distribution.

Folders	Files	Preview
Main folder	Base_de_Datos_PRPD	
Subfolders (Type of discharge)	Internal	
	Surface	
	Corona	
	noise	
Inside each subfolder (real/simulated/generated + (# of image))	real(1)	
	Sim(1)	
	gen(1)	

## 3 Data Description

**Fig. 1:**
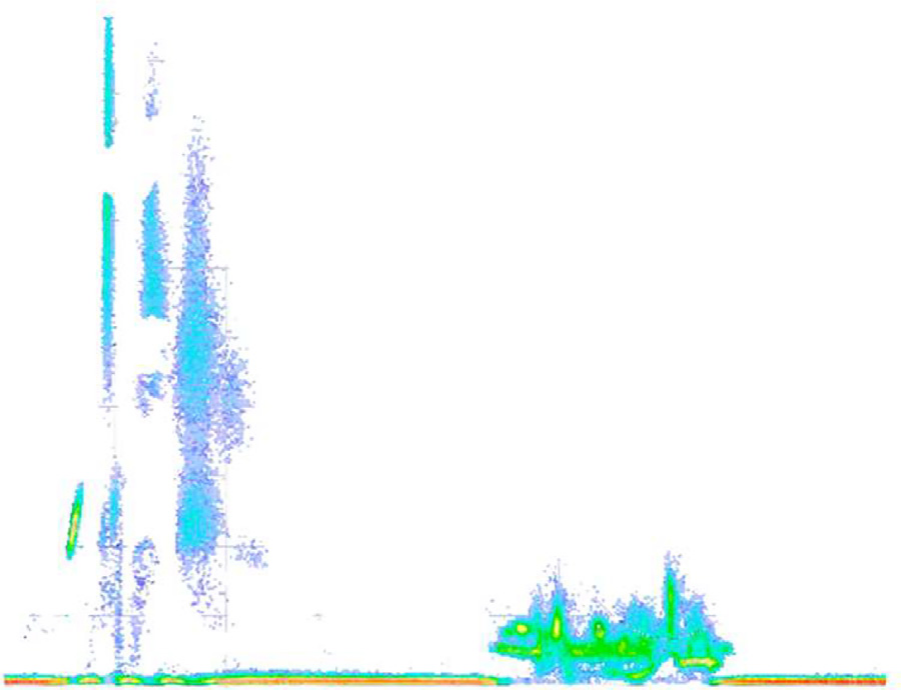
Corona-type defect captured in an Omicron PD simulator. *Source:* Own work.

The dataset encompasses a distribution that includes real images directly obtained from generators, images simulated using specialized equipment with manufactured electrodes, as well as images generated using data augmentation tools. The “Experimental Design, Materials, and Methods” section will provide detailed information on the data augmentation process for the acquired images. Additionally, the dataset includes images representing noise signals. These noise images have not undergone the data augmentation process; however, they offer an advantage in terms of classification and filtering when developing artificial intelligence systems for pattern recognition. The distribution of images and their quantity is presented in the following table:

This diversified approach to dataset composition ensures a holistic representation and comprehensive approach when developing and evaluating pattern recognition algorithms and artificial intelligence systems. The organization of the images is structured into folders as follows:

It is relevant to highlight that the images classified as “real” encompass both those obtained through measurements in generators and those captured in a partial discharge simulator. The partial discharge simulator, in this context, serves as a device that emulates the partial discharge process using an electrode. Furthermore, this device provides the capability to connect additional equipment to record and analyze the behavior of the detected anomaly. Fig. 1 is an image of a phase-resolved pattern of a corona-type defect mounted on the electrode simulator by Omicron Energy.

## 4 Experimental Design, Materials, and Methods

### 4.1 Images obtained from on-site measurements

The images obtained on-site were collected from various locations across the country, including hydroelectric and thermal power plants with generators having a nominal power greater than 12 MVA and voltages exceeding 6.6 kV. These sites were part of the field experience of some of the authors. This compilation of patterns does not contain any private information regarding the asset owners. In this dataset, only PRPD images are included, which could be acquired by another expert at any time. Each acquired image was rigorously obtained following the procedures outlined in the IEC 60270, IEC 60034-27, and IEEE 1434 standards.

**Fig. 2:**
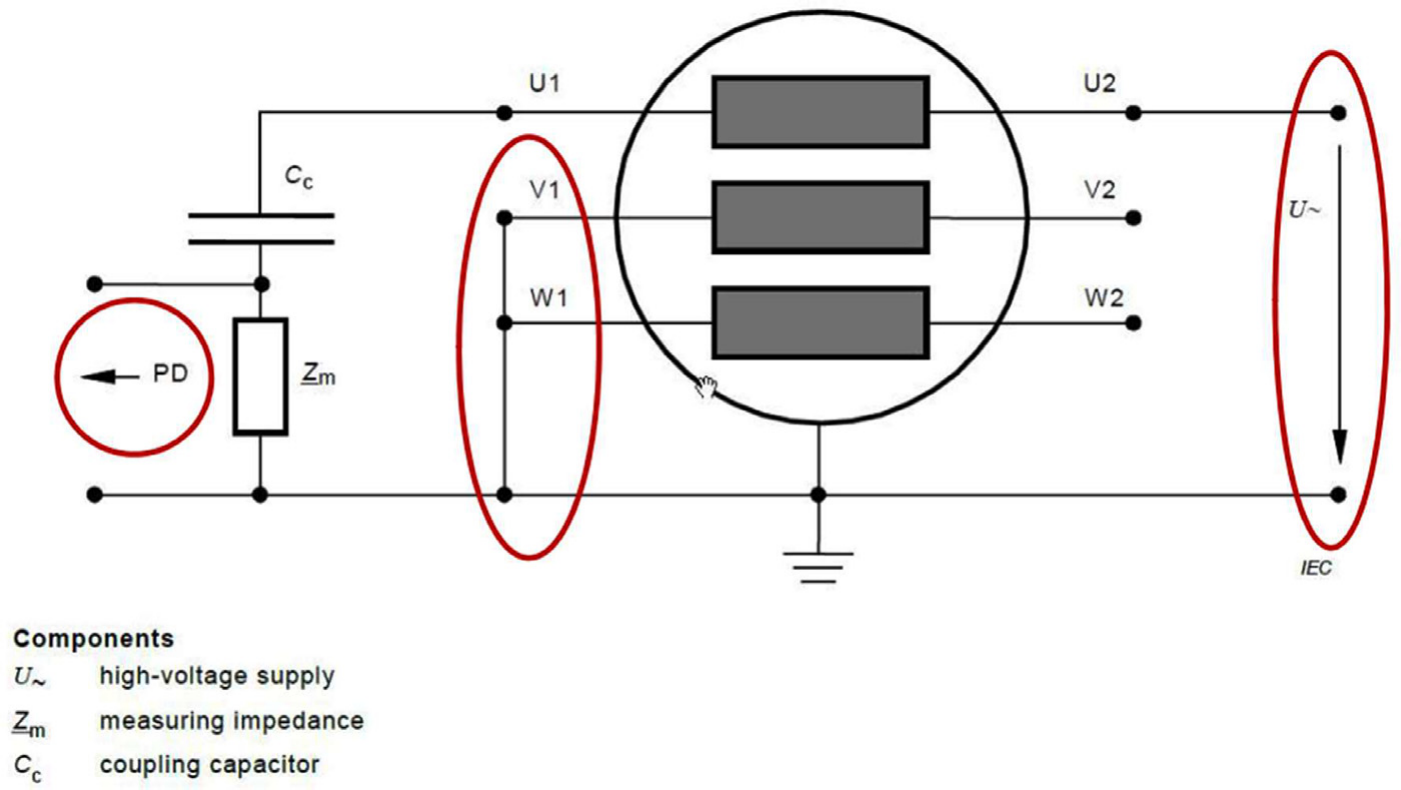
PD measurement connection circuit according to IEC 60034-27.

In accordance with the guidelines established by the IEC 60034-27 standard, the standard electrical measurement circuit designed for rotating machines was employed, with pulse detection carried out through a coupling system with capacitors, specifically 2 nF coupling capacitors. The connection of the measurement circuit, as detailed in Fig. 2, is followed by a calibration and normalization phase of the system to ensure the reliability and accuracy of the obtained measurements.

Normalization constitutes an essential procedure in the realm of partial discharge (PD) measurements. Given the intricate nature of the propagation of PD pulses, obtaining a direct measurement of the actual charge at the point of origin proves unfeasible. Consequently, the measurement of apparent charge is adopted, involving a calibration process, or more precisely, the normalization of the measurement.

The underlying purpose of calibration is to verify the measurement system's capability to accurately quantify the magnitude of the specified partial discharges. Simultaneously, the aim is to determine the calibration constant or scale factor (pC/mV) of the measurement system. This parameter is crucial as it establishes the relationship between the peak value of the waveform at the output of the detector system and the injected charge value by a short-duration current pulse calibrator, whose charge is known with certainty.

In this normalization process, the central frequencies of the filters and the corresponding bandwidths necessary for data acquisition are established. Table 4 details the generally employed central frequencies for conducting measurements on generators. It is noteworthy that, due to the variable nature of discharges, the same frequencies are not always used. Nevertheless, it is important to highlight that the IEEE 1434 standard specifies certain frequencies that should be considered in these procedures.

**Table 4:** Central frequencys, bandwidths common for PD.

Central frequency	Bandwidth
250 kHz	160 kHz
400 kHz	160 kHz
1 MHz	300 kHz

Thus, through the PD Suite software, the MPD 600 device, and the configuration and monitoring of each step, phase-resolved pattern captures were compiled for the generators listed in the file table as real images.

The verification of the type of partial discharge in generators requires the implementation of various specialized techniques. Partial Discharge Pattern Analysis (PRPD) is employed to visualize and assess temporally and spatially the discharges. Measurements of the partial discharge level provide a quantitative assessment of the quantity and intensity of the present discharges. Frequency analysis aids in identifying the nature and location of partial discharges. The use of advanced equipment, such as acoustic sensors and imaging systems, provides a more detailed evaluation. Furthermore, vacuum and impulse tests are applied to assess the generator's resistance to partial discharges under specific conditions. Continuous monitoring with data acquisition systems over time allows for the identification of patterns of partial discharges, contributing to preventive maintenance and enhancing the reliability of the generator system. These comprehensive techniques ensure a thorough verification of the presence, intensity, and location of partial discharges, crucial for accurate diagnosis and effective maintenance of electrical generators.

### 4.2 Images obtained from Omicron simulator

The images of phase-resolved PD patterns were simulated using a device developed by the company OMICRON called the PD simulator (a device not available for sale), which features three types of electrodes as seen in Image 2. Each electrode corresponds to a type of partial discharge fault: corona, internal, and surface, each with a defined behavior. Generation of these patterns is done using a source with a frequency of 200 Hz. The characteristics of the source and its behavior do not interfere with the comparison of images since PD patterns do not have defined axes for classification; classification is solely based on spatial distribution within the image. However, these axes serve to determine the magnitude or criticality of the PD and to indicate at which frequencies a high repeatability of the PD occurs. This information is used not only for classification but also to determine the criticality of the defect or fault.



Image 1 PD simulator. *Source:* Own

### 4.3 Images obtained through data augmentation

The term “Data Augmentation” (DA) refers to a technique that allows for the artificial generation of data by introducing perturbations to the original data. This increases diversity within the training dataset, making it suitable for effective supervised learning in artificial intelligence. Various approaches and techniques, such as circular shifting used in image classification with AI, as in [1,2], have been developed for this purpose.

In the development of this dataset, it was necessary to employ this technique for the reasons outlined in the “Value of the Data” section.

### 4.4 Data augmentation development

Once the classification of real and simulated images is complete, the Data Augmentation (DA) method is implemented. For this purpose, the Python programming library called “Augmentor” is employed. This library comprises a set of software tools designed for expanding image datasets, with a focus on providing operations commonly used in generating image data, specifically aimed at addressing challenges in the field of machine learning.

Initially, Augmentor incorporates a range of classes that facilitate the execution of conventional image manipulation functions, such as rotation or cropping. Additionally, the method encompasses a wide variety of practical functionalities, covering most essential procedures for efficiently expanding image datasets. The application of operations to images in Augmentor is carried out stochastically as they pass through a created pipeline, according to a user-defined probability value for each operation. Therefore, each operation has at least one probability parameter, which controls the likelihood of applying the operation to each image as it passes through the pipeline.

In this work, it is of utmost importance to carefully select the exact probability values, magnitude changes, pixel cuts, pixel enlargements, deformations, etc., as any operation that does not meet specific conditions can potentially compromise the integrity of an extracted pattern and, consequently, the reliability of the created database.

**Fig. 3:**
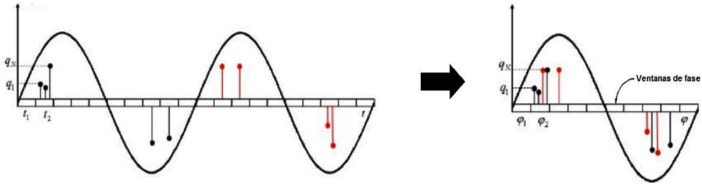
Summation of pulses in the Phase-Resolved Diagram of PD (Partial Discharge) [3].

To properly define the parameterization of the creation system, certain theoretical aspects in obtaining the Phase-Resolved Patterns (PRPD) must be considered, as follows:

A phase-resolved pattern of partial discharges or resolved phase diagram is the most commonly used analysis in partial discharge diagnosis, as there is a strong relationship between the pattern's shape and the type of defect causing it [1]. In the diagram, the magnitude of the PD pulse “q” in pC (picocoulombs) or mV (millivolts) on the Y-axis is plotted against its phase of occurrence ∅ in degrees on the X-axis, and these are superimposed for each cycle of the sinusoidal injection wave applied to the test object, as shown in Fig. 3.

In order for the magnitude value of a pulse, measured in volts or coulombs, to be represented in a Phase-Resolved Diagram, two general conditions must be met:

1. ***Increase in Voltage:*** The voltage within the electric field of the cavity or void must exceed the dielectric breakdown voltage, creating an avalanche of electrons. This results in a slight decrease in the voltage value, and therefore, a negative magnitude peak with respect to the half-cycle in which it occurred.
2. ***Recording of Phase Value:*** The phase value or occurrence, measured in degrees or milliseconds, at which this voltage decrease occurs must be recorded. These values are referenced between 0° and 360° or 0 and 16.6 ms, which is the complete duration of one cycle of the sinusoidal injection wave.

The second condition mentioned above, in particular, is the most crucial condition for generating augmented data through programming. This is because the pulse value or magnitude, along with its repeatability, serves as a reference point for the criticality of the discharge. It provides information about how high and severe the discharge is but does not indicate the classification of the discharge type as “internal, corona, or surface.” On the other hand, the combination of the magnitude value with its phase value does provide classification information since it depends on the half-cycle in which the discharge occurs or its distribution within a range of degrees, which relates to its location.

**Fig. 4:**
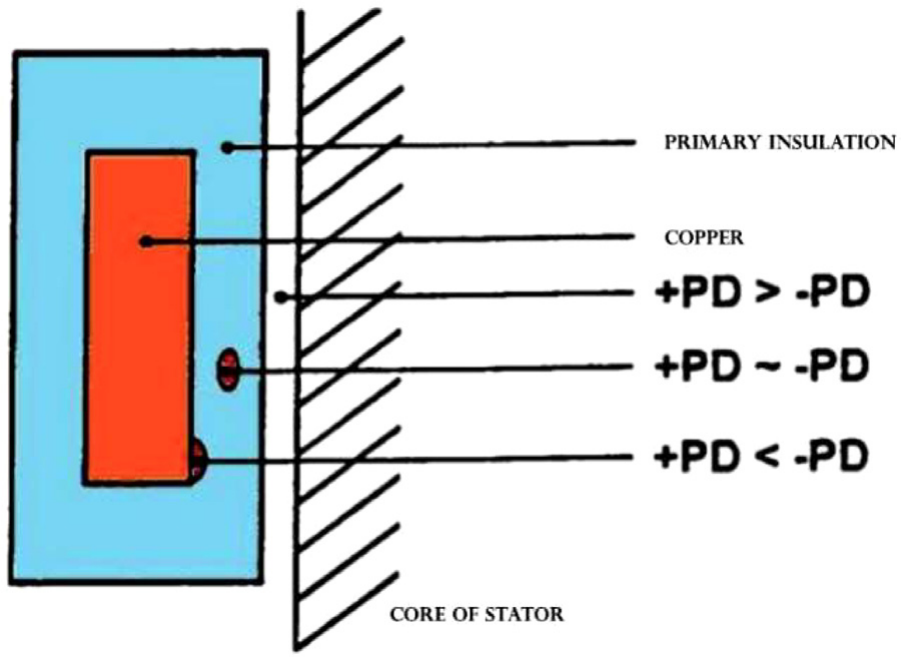
Location of Internal PDs According to their Source Phase: J. Toro. International Seminar on Electrical Testing. [2].

In Fig. 4, +PD corresponds to pulses generated in the first quarter of the waveform cycle (rise), while -PD values are generated in the second quarter of the sinusoidal waveform cycle (fall). If either of these pulse types predominates, it indicates an internal location near the conductor, the core, or between them [3].

**Fig. 5:**
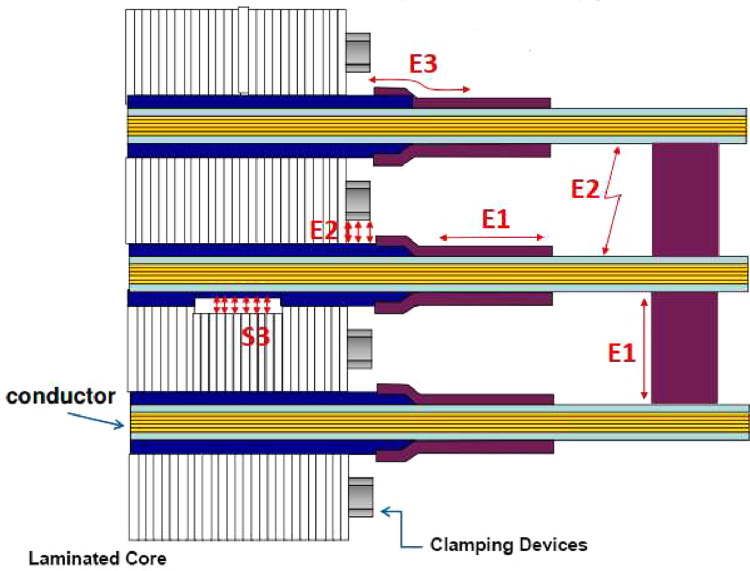
Location of Surface Discharges. *Source:* Omicron PD Seminar.

In the case of surface discharges, negative pulses predominate, and they typically appear as increasing clouds between 30 and 40 degrees of the corresponding positive half-cycle, especially in cases of tracking on contaminated surfaces (E1, E3 in Fig. 5). There are also horizontal clouds that have both positive and negative pulses with similar repetition rates and magnitudes, spanning the entire half-cycle of the waveform and changing their polarity value in the next half-cycle. These tend to be more critical in machine diagnosis as they appear at a level even perceptible in terms of noise and visual observation (E2 in Fig. 5) [4].

The above demonstrates that generating a phase movement concentration of several points in a PRPD image not only requires expertise but also a thorough understanding of the measurement method to calculate the parameterization values with which new images will be generated. This is essential to avoid significant distortion of the original PRPDs, which can lead to incorrect classification and introduce significant biases into AI results.

### 4.5 Parameterization of augmentor

An example of Augmentor parameterization is presented in Table 5, which shows different levels of distortions. It illustrates that not all distortions fulfill the condition of preserving the relevant information of the PRPDs while slightly altering their shape. Instead, some distortions involve sharp cuts and abrupt movements that completely change the nature of the initial pattern.

**Table 5:** Deformation stages in data augmentation method source: own compilation.

Deformation stages	
Original	Deformation 1 - Simple distortion
	
Deformation 2 - Pixel stretching	Deformation 3 - Tilt shearing (Information loss occurs)
	
Deformation 4 - Originality loss	

The permissible images generated by data augmentation extend up to the second deformation. Beyond that point, many data points can become mixed and move far from their initial phase, leading to a selection bias and creating false-negative relationships in image classification used in supervised learning.

The parameters used to generate the database are as follows:

**Gaussian Deformation:** This is used to introduce random slight directional movements in both axes of the pixels in the sample. This ensures that the initial characteristics are not lost, and the size is preserved. The probability function dictates that results are reproduced with certain values according to the specifications.



**Code Line 1**

• **Scaling and Vertical Distortion:** This operation zooms in on the images based on the factor selected by the programmer.

**Code Line 2**

• **Shearing**: It creates diagonal cuts with a specified angle value.

**Code Line 3**

• **Vertical Axis Reflection**: This operation generates a mirrored image by flipping the quadrants in 3 stages.

**Code Line 4**

• **Horizontal Axis Reflection**: It generates a mirrored image by flipping the quadrants in 6 stages.

**Code Line 5**

### 4.6 Data augmentation results

**Fig. 6:**
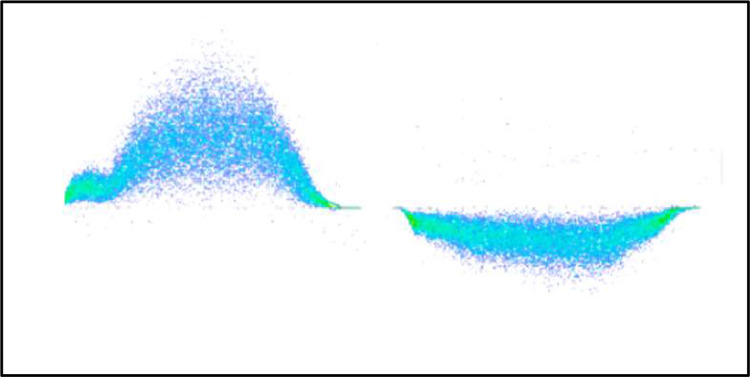
Original image for Analysis and Augmentor Deformation *Source:* Database Measurement.

The outcomes of image processing are elucidated in Table 5, which comprises the resulting deformations resulting from the aforementioned lines of code and the execution on the Augmentor's programmable core. Fig. 6 includes an original image to allow the reader to observe the disparities outlined in Table 6, where the rationale behind each result is also explained.

**Table 6:** Data augmentation results.

Type of deformation	Result	Acceptance criterion
Pattern amplification by sections		Registered DP point manifestations are not lost; their distribution merely changes in controlled areas, ensuring that the original pattern's shape remains intact, preserving its classification.
Frequency-domain DP sampling change		Moving DP point manifestations along the horizontal axis does not alter their shape, only the frequency at which they were manifested. This is synonymous with changing the value of insulation capacitance while keeping the same deformation caused by DP (internal, surface, or corona).
Slight magnitude DP enlargement		Vertical stretching increases the magnitude of DP without altering any pattern behavior, only expanding the area vertically.
Polarity change		Polarity changes are introduced to anticipate a phase shift in frequency of 90° or greater, without losing pattern characteristics.
Scale factor change		Zooming in on the image expands the sample area without compromising the distribution of points generated by DP.
Slight DP manifestation movement in both polarities		Using a programmable shear, the image can be distorted at angles ranging from 0 to 25 degrees, altering both frequency and magnitude, albeit with low probability in the code. This helps reduce selection bias for measurements outside the database by a significant percentage.

## Ethical Statements

This dataset does not contain information involving humans, animal experiments, or data collection from social media platforms.

## CRediT Author Statement

Juan David Zorrilla Henao: Conceptualization, methodology, software, research.

Hermes Alejandro Tenorio Tamayo: Writing - Original draft preparation, research.

José Alejandro Jiménez Segura: Software validation, resources.

Harold Diaz: Supervision.

Alejandro Paz: Supervision, Writing - Review and Editing.

## Declaration of Competing Interest

The authors declare that they have no competing financial interests or known personal relationships that could have influenced the work reported in this paper.

## Data Availability

Images of resolved phase patterns of partial discharges in electric generators (Original data) (Mendeley Data)
